# Different profiles of neurocognitive impairment in patients with hepatitis B and C virus infections

**DOI:** 10.1038/s41598-022-14736-3

**Published:** 2022-06-23

**Authors:** Chun-Hsiang Tan, Meng-Chia Chang, Wei-Fang Tsai, Wan-Long Chuang, Jee-Fu Huang, Zu-Yau Lin, Chia-Yen Dai, Ming-Lun Yeh, Chi-Ting Li, Rwei-Ling Yu

**Affiliations:** 1 Department of Neurology, Kaohsiung Medical University Hospital, Kaohsiung Medical University, Kaohsiung, Taiwan; 2 Graduate Institute of Clinical Medicine, College of Medicine, Kaohsiung Medical University, Kaohsiung, Taiwan; 3 Institute of Behavioral Medicine, College of Medicine, National Cheng Kung University, Tainan, Taiwan; 4 M.Sc. Program in Tropical Medicine, College of Medicine, Kaohsiung Medical University, Kaohsiung, Taiwan; 5 Hepatobiliary Division, Department of Internal Medicine, Kaohsiung Medical University Hospital, Kaohsiung Medical University, Kaohsiung, Taiwan; 6 Department of Psychology, Kaohsiung Medical University, Kaohsiung, Taiwan

**Keywords:** Neuroscience, Gastroenterology, Neurology

## Abstract

The direct impact of chronic hepatitis B and hepatitis C on neurocognition remains elusive due to the frequent comorbidities, and the domains of the neurocognitive functions affected have rarely been investigated comprehensively. We cross-sectionally assessed the neurocognitive functions of the individuals with chronic hepatitis B, chronic hepatitis C, treated chronic hepatitis C with a sustained virologic response, and their healthy control counterparts. Laboratory examinations were used to investigate the impact of inflammation on neurocognition, exclude the medical conditions that could interfere with neurocognition assessment, and assess liver function and fibrotic severity of the liver of the participants. This study found the detrimental impact of chronic hepatitis B on language and executive functions. In contrast, individuals with chronic hepatitis C showed deficits in executive functions, psychomotor speed, memory, and attention. Successful elimination of hepatitis C resulted in improved liver function, but not neuropsychological test performance. Moreover, erythrocyte sedimentation rate level was found to mediate the deficits in the attention of individuals with chronic hepatitis C. These results demonstrate the neurocognitive deficits and the difference in the profiles of neurocognitive deficits in individuals with chronic hepatitis B and chronic hepatitis C. Our study also provided results suggesting the mediation by systemic inflammation on the attention deficit in individuals with chronic hepatitis C.

## Introduction

According to the global hepatitis report 2017 by the World Health Organization, in 2015, the global prevalence of HBV and HCV infection in the general population was estimated to be 3.5% and 1%, respectively^1^. In addition to the life-threatening complications, including cirrhosis and hepatocellular carcinoma, both HBV and HCV cause extrahepatic manifestations. More importantly, these extrahepatic manifestations cause an impaired quality of life and economic burden on patients with viral hepatitis^2^. Among the extrahepatic manifestations, the neuropsychiatric symptoms found in individuals with HCV have been reported previously. However, with depression, cirrhosis, thyroid disorders, cerebrovascular diseases, and end-stage renal diseases as common comorbidities associated with HCV and significantly interfering with the accurate evaluation of neurocognitive functions, the inclusion of patients with the common comorbidities associated with HCV in the previous studies prevents a clear demonstration of the direct effect of HCV infection on neurocognitive function changes. In addition, while the negative impact of HCV infection on neurocognitive functions has been shown across several studies, the investigation into the effects of HBV on cognition has rarely been reported. As HBV has an even higher prevalence than HCV and is still without the treatment for cure, an investigation to understand the long-term impact of HBV on neurocognitive functions is warranted.

Furthermore, several mechanisms through which HCV causes neurocognitive deficits have been proposed, but a definite conclusion remains to be explored due to the limited evidence available. Meanwhile, although the treatment regimens for HCV infection have advanced significantly, whether the neurocognitive deficit of patients with HCV is reversible and whether successful elimination of HCV infection causes improvement in neurocognitive functions remains unknown. Finally, as the concept of "neurocognitive functions" is a broad term and covers a wide range of complex mental processes, the elucidation of the neurocognitive functions attributable to specific conditions requires comprehensive assessments covering different domains of each ability area and a careful combination of tasks to reveal patterns of performance^3^. However, a thorough evaluation of the neurocognitive functions covering all the major neurocognitive domains of patients with chronic viral hepatitis has rarely been investigated.

Taiwan used to have a high prevalence of chronic HBV and HCV. The prevalences of HBV and HCV among the general Taiwanese population were previously reported to be around 15–20% and 4.1%, respectively^4,5^. The prevalence was even reported to be up to 17.2% in Kaohsiung County, the southern part of Taiwan^6^. Although the prevalences of HBV and HCV have significantly decreased in recent years, the impact of chronic viral hepatitis on the generation born before the nationwide HBV vaccination program in 1986 has been long-lasting and remained not entirely clear. The situation is similar for those infected with HCV before the wide availability of effective treatment against HCV. Therefore, in the current study, we comprehensively evaluated the neurocognitive functions (summarized in Table 1) of healthy participants without viral hepatitis and those with chronic HBV or HCV to understand the long-term impact and potential mechanisms of the neurocognitive deficit caused by chronic HBV and HCV infection.

**Table 1:** Summary of the neuropsychological tests used for the assessment of the major cognitive domains.

Domain	Neuropsychological tests
Executive function	Similarities of Wechsler Adult Intelligence Scale-IV
	Matrix Reasoning of Wechsler Adult Intelligence Scale-IV
	Information on Wechsler Adult Intelligence Scale-IV
	Letter-Number Sequencing of Wechsler Adult Intelligence Scale-IV
	Semantic association of category verbal fluency
	Trial 2 of Color Trails Test
	Modified Wisconsin Card Sorting Test
Memory	Logical Memory-I&II of Wechsler Memory Scale-III
	Visual Reproduction-I&II of Wechsler Memory Scale-III
Psychomotor speed	Trial 1 of Color Trails Test
	Digit Symbol Substitution of Wechsler Adult Intelligence Scale-IV
	Symbol Search of Wechsler Adult Intelligence Scale-IV
Visuospatial function	Block Design of Wechsler Adult Intelligence Scale-IV
Attention	Stroop Color and Word Test
	Digit Span of Wechsler Adult Intelligence Scale-IV
	Paced Auditory Serial Addition Test
	Serial seven of the Mini-Mental State Examination
Language	Naming, Repetition, Verbal Comprehension, and Writing of Mini-Mental State Examination

## Results

### Patients with chronic viral hepatitis showed different profiles of neurocognitive deficit across different domains

To examine whether chronic viral hepatitis causes neurocognitive deficits and assess which neurocognitive domains were affected, the participants were divided into four groups: healthy volunteers, participants with HBV, participants carrying HCV, and participants with a sustained virologic response (SVR) after HCV treatment. All the participants with HBV had a chronic infection as determined by HBsAg positivity for six months or more or the presence of HBV DNA by polymerase chain reaction six months or more after the initial HBsAg positivity. The number of participants in the phase of HBeAg positive chronic infection, HBeAg positive chronic hepatitis, HBeAg negative chronic infection, and HBeAg positive chronic hepatitis were 1, 7, 27, and 36, respectively, and 43 of the participants with HBV were taking antiviral medication against HBV. All the participants with untreated HCV had chronic HCV as determined by the persistent presence of HCV RNA by polymerase chain reaction for six months or more until the neuropsychological test. The definition of SVR was based on the absence of HCV RNA by polymerase chain reaction six months after the end of anti-HCV therapy before the assessment with the neuropsychological test. The demographic data, including age, sex, education, hypertension (HTN), diabetes mellitus (DM), signs of polyneuropathy, erythrocyte sedimentation rate (ESR), C-reactive protein (CRP), alanine transaminase (ALT), and FIB-4 index of the participants within each study group are shown in Table 2. There was no significant difference in the percentage of participants with HTN, DM, or signs of polyneuropathy observed among the different study groups. There were significant differences in age, sex ratio, education years, ESR, and ALT between the study groups. The difference in age and sex ratio between the different study groups may be due to the difference in the epidemiological profile of HBV and HCV among Taiwanese, and the difference in the education years may be secondary to the difference in age and sex ratio. As ESR is closely associated with age and sex, which are variables significantly different between the study groups, we used Quade's test to compare ESR and CRP levels between the study groups to eliminate age and sex's confounding effect. After controlling the effect of age and sex, no statistically significant difference was observed between the study groups, and the results are shown in supplementary table 1. Although the ALT level of the untreated HCV group is significantly higher than those of the HBV and SVR group, the mean values of the ALT in the three groups were all below the twofold upper limit of the normal ALT reference range, indicating that most of the patients are not in the acute exacerbation phase. Furthermore, there was no statistically significant difference in the FIB-4 among the study groups. The mean values of the FIB-4 in the three groups were all lower than 3.25 (the value over which can be suggestive of significant cirrhosis), indicating that most of the participants recruited did not have significant cirrhosis.

**Table 2:** Demographic characteristics in the study groups.

	Control	HBV	HCV	SVR	*p* value
Number	40	71	23	65	–
Age (years)	65.00 ± 6.320	59.00 ± 7.051	62.00 ± 7.793	62.05 ± 9.308	0.002
Sex(M/F)	9/31	52/19	3/20	28/37	< 0.001
Education (years)	13.15 ± 3.984	13.27 ± 3.321	10.39 ± 3.144	11.37 ± 3.773	< 0.001
HTN (Y/N)	10/30	24/47	6/17	22/43	0.698
DM (Y/N)	4/36	10/61	2/21	13/52	0.413
Signs of PN	–	10/61	1/22	16/49	0.057
ESR (mm/h)	–	11.42 ± 8.920	23.61 ± 17.026	16.59 ± 13.814	0.001
CRP (mg/L)	–	1.45 ± 2.853	3.23 ± 6.376	1.48 ± 2.695	0.482
ALT (IU/L)	–	31.03 ± 22.398	62.61 ± 47.548	26.05 ± 12.199	0.002
ALT > 80 IU/L (Y/N)	–	3/68	7/16	0/65	< 0.001
FIB-4 index	–	1.88 ± 0.894	2.82 ± 2.130	2.14 ± 1.105	0.211

Laboratory reference ranges for each of the tests: ESR: 0–15 mm/h for men and 0–20 mm/h for women; CRP: < 5 mg/L; ALT: 10–40 IU/L; FIB-4 > 3.25 had a positive predictive value for a significant fibrosis (F3-F4) of 82.1% with a specificity of 98.2%^60^.

*ALT* alanine aminotransferase, *CRP* C reactive protein, *DM* diabetes mellitus, *ESR* erythrocyte sedimentation rate; *HBV* hepatitis B virus, *HCV* hepatitis C virus, *HTN* hypertension, *PN* polyneuropathy, *SVR* sustained virologic response.

The profiles of the neurocognitive functions of each study group's participants are shown in Table 3, and the existence of the differences in neurocognitive functions between different study groups was examined with the Kruskal–Wallis test. Statistically significant differences between the study groups were noted in (1) both the immediate and delayed recall of logical memory and the immediate recall of visual production in Wechsler Memory Scale-III (WMS-III); (2) full-scale intelligence quotient, verbal comprehension index, perceptual reasoning index, similarities, information, block design, matrix reasoning, digit span total score, longest digit span forward, digit symbol substitution, and symbol search of Wechsler Adult Intelligence Scale-IV (WAIS-IV); (3) Paced Auditory Serial Addition Test (PASAT); (4) semantic association of category verbal fluency; (5) trial 2 of Color Trails Test (CTT). The results of the post-hoc analysis identifying the significant between-group differences with the Dunn's test after the Kruskal–Wallis test are shown in Supplementary Table 2.

**Table 3:** Neurocognitive functions of the study groups.

			Control		HBV		HCV		SVR		Kruskal–Wallis *p* value
			Mean	SD	Mean	SD	Mean	SD	Mean	SD	
Mini Mental Status Examination	Serial 7		4.450	0.846	4.507	0.715	4.304	1.146	4.400	0.932	0.986
	Language		4.825	0.385	4.732	0.446	4.826	0.491	4.892	0.312	0.105
Wechsler Memory Scale-III	Logical Memory	Immediate recall	32.675	11.237	33.648	9.744	29.130	12.618	28.446	10.980	0.033*
		Delayed recall	19.325	8.871	20.577	7.985	17.652	7.377	16.231	8.087	0.012*
	Visual Reproduction	Immediate recall	76.525	16.045	75.746	13.000	71.435	17.291	67.308	17.121	0.009*
		Delayed recall	52.050	22.160	54.859	20.517	42.217	23.522	45.815	25.079	0.046*
Wechsler Adult Intelligence Scale-IV	Full Scale Intelligence Quotient		106.750	16.442	103.254	13.964	95.217	13.359	97.892	14.250	0.003*
	Verbal Comprehension Index		110.675	16.665	105.352	14.571	96.130	14.772	98.754	14.850	< 0.001*
	Perceptual Reasoning Index		105.400	13.513	102.197	14.423	94.000	11.954	97.169	13.129	0.002*
	Working Memory Index		100.675	15.628	102.282	13.407	95.478	12.685	97.954	14.068	0.117
	Processing Speed Index		103.050	15.081	102.634	13.192	98.870	15.185	100.400	13.862	0.484
	Similarities		20.650	6.941	20.197	5.749	15.087	5.204	16.508	6.874	< 0.001*
	Information		12.150	5.342	11.577	4.889	8.565	5.822	9.246	5.477	0.003*
	Block Design with No Time Bonus		33.200	8.112	33.549	8.037	27.478	7.937	30.138	8.344	0.005*
	Matrix Reasoning		15.275	5.416	14.296	5.041	12.217	4.253	12.831	5.134	0.041*
	Letter-Number Sequencing		16.150	5.162	17.915	4.423	15.870	4.920	16.554	4.864	0.128
	Digit Span		25.450	5.835	27.239	5.577	23.565	4.021	24.092	6.463	0.005*
	Longest Digit Span Forward		7.200	1.091	7.690	1.129	6.739	1.096	7.000	1.335	0.001*
	Longest Digit Span Backward		4.700	1.588	5.042	1.439	4.652	1.112	4.692	1.520	0.656
	Digit Symbol Substitution		57.625	15.686	63.704	15.235	56.652	17.393	56.369	19.989	0.029*
	Symbol Search		25.325	8.216	29.155	7.365	24.174	8.773	26.323	8.885	0.025*
Paced Auditory Serial Addition Test			70.150	18.647	76.197	13.216	63.304	19.170	67.569	21.708	0.027*
Modified Wisconsin Card Sorting Test	Number of Categories Completed		5.350	1.777	5.352	1.821	4.391	2.251	4.723	2.204	0.151
	Number of Perseverative Errors		4.050	4.820	3.465	4.475	5.609	5.088	5.031	6.357	0.139
Stroop Color and Word Test	Interference Score		−3.843	6.910	−3.957	8.643	-3.660	9.459	-5.246	8.409	0.690
Semantic Association of Category Verbal Fluency	Total		39.275	8.732	34.366	8.086	38.478	7.733	36.985	9.048	0.029*
Color Trails Test	Trial 1		53.327	18.783	45.926	21.650	48.680	20.668	51.033	23.313	0.076
	Trial 2		113.595	38.821	97.369	38.505	118.157	44.026	118.783	59.139	0.017*

*HBV* hepatitis B virus, *HCV* hepatitis C virus, *HTN* hypertension, *SD* Standard deviation, *SVR* sustained virologic response; *Statistically significant.

However, as shown in Table 2, significant differences exist in the demographic characteristics between the study groups. Thus, the significant differences in the task performance, as shown in Table 3 and Supplementary Table 2, may be secondary to the differences in the demographic characteristics between the study groups, such as age, sex, and education. To eliminate the effect of the potential confounding factors, we used Quade's test and set age, sex, and education years as covariates to compare the participants' neurocognitive functions within each study group. As shown in Table 4, after controlling the potential effect of age, sex, and education years with the Quade test, patients with HBV showed significantly worse performance in verbal comprehension index, information, and matrix reasoning of WAIS-IV than their healthy control counterparts. Besides, patients with HBV showed significantly worse performance in the language function than those in the SVR group (with a borderline significant difference compared with the HCV group). These results suggest the negative impact of HBV on the neurocognitive domains of language function and executive function.

**Table 4:** Comparison of neurocognitive functions between the study groups with age, sex and education corrected.

			Control-HBV		Control-HCV		Control-SVR		HBV-HCV		HBV-SVR		HCV-SVR	
			Quade's test											
			F	P	F	P	F	P	F	P	F	P	F	P
Mini Mental Status Examination	Serial 7		0.064	0.801	0.643	0.426	1.511	0.222	1.246	0.267	0.456	0.501	0.000	0.985
	Language		1.413	0.237	0.585	0.448	1.508	0.222	4.901	0.029*	9.862	0.002*	0.068	0.794
Wechsler Memory Scale-III	Logical Memory	Immediate recall	0.013	0.910	0.008	0.928	1.109	0.295	0.778	0.380	3.698	0.057	0.042	0.839
		Delayed recall	0.052	0.821	0.000	0.991	1.678	0.198	0.335	0.564	5.349	0.022*	0.393	0.533
	Visual Reproduction	Immediate recall	3.207	0.076	0.962	0.331	7.812	0.006*	1.415	0.237	1.047	0.308	1.526	0.220
		Delayed recall	0.710	0.401	7.083	0.010*	2.596	0.110	0.037	0.848	0.057	0.812	0.950	0.332
Wechsler Adult Intelligence Scale-IV	Full Scale Intelligence Quotient		2.069	0.153	1.756	0.191	0.902	0.345	0.516	0.474	0.215	0.643	0.047	0.828
	Verbal Comprehension Index		4.714	0.032*	2.103	0.153	5.181	0.025*	0.308	0.580	0.528	0.469	0.342	0.560
	Perceptual Reasoning Index		3.676	0.058	5.889	0.019*	4.358	0.040*	1.012	0.317	0.014	0.906	0.153	0.696
	Working Memory Index		0.598	0.441	0.166	0.685	0.021	0.885	0.000	0.990	0.144	0.705	0.024	0.876
	Processing Speed Index		0.812	0.370	0.004	0.952	0.750	0.389	0.041	0.840	0.192	0.662	0.000	0.998
	Similarities		0.501	0.481	5.225	0.026*	1.919	0.169	0.893	0.347	1.774	0.185	0.099	0.754
	Information		4.140	0.045*	1.215	0.275	3.105	0.081	0.957	0.331	0.042	0.838	0.297	0.587
	Block Design with No Time Bonus		1.076	0.302	3.480	0.068	2.564	0.113	0.062	0.804	0.014	0.906	0.098	0.755
	Matrix Reasoning		6.789	0.011*	3.907	0.053	2.931	0.090	2.240	0.138	0.394	0.531	0.088	0.767
	Letter-Number Sequencing		0.722	0.398	0.018	0.893	0.457	0.501	0.111	0.740	0.448	0.504	0.144	0.706
	Digit Span		0.012	0.912	0.619	0.435	0.484	0.488	0.732	0.394	1.500	0.223	0.037	0.848
	Longest Digit Span Forward		1.444	0.232	1.302	0.259	0.126	0.723	2.745	0.101	3.393	0.068	1.512	0.222
	Longest Digit Span Backward		0.005	0.942	0.077	0.783	0.436	0.511	0.465	0.497	0.069	0.794	0.001	0.972
	Digit Symbol Substitution		1.656	0.201	0.018	0.895	0.511	0.476	0.007	0.933	0.836	0.362	0.024	0.877
	Symbol Search		1.866	0.175	0.475	0.494	0.922	0.339	1.460	0.230	0.006	0.936	1.048	0.309
Paced Auditory Serial Addition Test			0.029	0.864	0.380	0.540	0.067	0.796	0.102	0.750	0.000	0.998	0.366	0.547
Modified Wisconsin Card Sorting Test	Number of Categories Completed		0.068	0.795	1.443	0.235	0.085	0.771	0.002	0.967	0.122	0.728	0.181	0.671
	Number of Perseverative Errors		0.033	0.856	1.017	0.318	0.046	0.831	0.026	0.873	0.386	0.535	0.767	0.384
Stroop Color and Word Test	Interference Score		1.243	0.268	0.029	0.866	0.739	0.392	2.425	0.123	0.002	0.960	0.970	0.327
Semantic Association of Category Verbal Fluency	Total		3.771	0.055	0.038	0.845	1.050	0.308	0.740	0.392	0.966	0.327	0.023	0.879
Color Trails Test	Trial 1		0.029	0.864	4.579	0.037*	1.685	0.197	0.353	0.554	0.000	0.984	0.992	0.322
	Trial 2		0.087	0.769	0.273	0.603	0.041	0.840	0.093	0.761	0.311	0.578	0.050	0.823

*HBV* hepatitis B virus, *HCV* hepatitis C virus, *HTN* hypertension, *SD* Standard deviation, *SVR* sustained virologic response.

The F values stand for the variations between the sample means divided by the variation within the samples, derived from the Quade test (one-way ANOVA after ranking the dependent variables and the covariates).

Meanwhile, the patients with HCV showed significantly worse performance in perceptual reasoning and similarities of WAIS-IV and the trial 1 of CTT than the healthy control counterparts. The results suggest that HCV causes a deficit in executive functions and psychomotor speed. For the SVR group, the patients showed significantly worse performance in the immediate recall of visual production in WMS-III and verbal comprehension index of WAIS-IV than their healthy control counterparts. Compared with the patients with HBV, the SVR group still showed worse performance in both the immediate and delayed recall of the logical memory of WMS-III and shorter longest digit span forward. The results indicate that HCV patients have deficits in memory and attention, and the worse performance in memory and attention can still be observed in those after successful treatment of HCV obtaining SVR. No statistically significant difference was observed when comparing the HCV patients with those whose HCV had been treated and obtained SVR. The results suggest that successful HCV treatment does not significantly improve the neurocognitive function deficit of individuals with viral hepatitis. However, a significant improvement in hepatic injury was observed, as evidenced by the ALT level.

### The neurocognitive deficit in individuals with viral hepatitis seems to be mediated by systemic inflammation, and attention is the most sensitive neurocognitive domain affected

In addition, we hypothesize that the systemic inflammation induced by HCV may be the mediator for developing neurocognitive deficits, which was examined with a regression-based analysis carried out with PROCESS macro on SPSS. The mediation effects of ESR and CRP on the neurocognitive functions affected by HCV compared with HBV are shown in Table 5 and supplementary table 3, respectively. As shown in Table 5, the significant mediation by ESR on the neurocognitive functions affected by HCV is demonstrated in serial 7 of Mini-Mental State Examination (MMSE), full-scale intelligence, working memory, block design, letter-number sequencing, and digit span of WAIS-IV, PASAT, and interference score of Modified Wisconsin Card Sorting Test (M-WCST). The statistically significant mediation by ESR in serial 7 of MMSE and working memory and digit span of WAIS-IV is still observed after correcting the potential confounders, including age, sex, and education years. These results suggest that the neurocognitive function deficit found in individuals with HCV is mediated partially through systemic inflammation, and attention is the most sensitive domain to the detrimental effect of systemic inflammation. Besides, the impact of systemic inflammation on neurocognitive functions is more closely associated with mechanisms affecting ESR, but not CRP.

**Table 5:** Mediation analysis of neurocognitive functions between the study groups by ESR.

			Mediation Analysis							
			No Covariates				Adjusted for age, sex, education, and metabolic syndrome			
			Effect	LLCI	ULCI	Sig.	Effect	LLCI	ULCI	Sig.
Mini Mental Status Examination	Serial 7		−0.1477	−0.3565	−0.0259	*	−0.0813	−0.2311	−0.0014	*
	Language		0.0036	−0.0378	0.0429	–	0.0079	−0.0155	0.0402	–
Wechsler Memory Scale-III	Logical Memory	Immediate recall	−0.7262	−2.2011	0.5519	–	−0.2989	−1.1943	0.5695	-
		Delayed recall	−0.5020	−1.5748	0.3268	–	−0.1728	−0.7694	0.3681	-
	Visual Reproduction	Immediate recall	−0.8457	−3.4606	0.8677	–	0.1751	−0.9251	1.1568	-
		Delayed recall	−0.1553	−3.2006	2.1475	–	0.8368	−0.4846	2.5532	-
Wechsler Adult Intelligence Scale-IV	Full Scale Intelligence Quotient		−1.6078	−4.2090	−0.0180	*	−0.5167	−1.6651	0.2363	–
	Verbal Comprehension Index		−1.6243	−4.4283	0.0956	–	−0.4347	−1.3955	0.1851	–
	Perceptual Reasoning Index		−1.1902	−3.4378	0.1957	–	−0.1287	−1.0475	0.7795	–
	Working Memory Index		−1.7786	−3.9999	−0.3869	*	−0.8216	−2.1997	0.0168	–
	Processing Speed Index		−0.5136	−2.6871	1.0397	–	−0.2566	−1.4146	0.7271	–
	Similarities		−0.6467	−1.7184	0.0021	–	−0.1446	−0.5734	0.1713	–
	Information		−0.5222	−1.4945	0.0786	–	−0.0526	−0.3581	0.1916	–
	Block Design with No Time Bonus		−1.2294	−2.5768	−0.3194	*	-0.3114	−0.9491	0.1825	–
	Matrix Reasoning		−0.1350	−0.8128	0.3978	–	0.1695	−0.0952	0.5398	–
	Letter-Number Sequencing		−0.5825	−1.4799	−0.0550	*	-0.1830	−0.6872	0.1066	–
	Digit Span		−0.7064	−1.5149	−0.1711	*	-0.2581	−0.6503	0.0151	–
	Longest Digit Span Forward		−0.1439	−0.3064	−0.0319	*	-0.0742	−0.1959	−0.0015	*
	Longest Digit Span Backward		−0.1223	−0.2993	−0.0021	*	-0.0476	−0.1549	0.0179	–
	Digit Symbol Substitution		−0.0089	−3.6601	3.2918	–	0.6509	−1.0935	2.6638	–
	Symbol Search		−0.6389	−1.9029	0.1780	-	−0.1039	−0.6516	0.3770	–
Paced Auditory Serial Addition Test			-2.3148	−5.2882	−0.2055	*	-0.6563	−2.2325	0.7175	−
Modified Wisconsin Card Sorting Test	Number of Categories Completed		−0.0787	−0.2977	0.1653	-	0.0117	−0.1058	0.1932	–
	Number of Perseverative Errors		0.1363	−0.3782	0.7552	–	−0.0228	−0.4168	0.3629	–
Stroop Color and Word Test	Interference Score		−0.7432	−1.6984	−0.0259	*	−0.3016	−1.0052	0.1458	–
Semantic Association of Category Verbal Fluency	Total		−0.4475	−1.6114	0.5286	–	−0.3943	−1.1921	0.2109	–
Color Trails Test	Trial 1		0.3375	−1.5488	2.3743	–	−0.4559	−2.1697	0.7837	–
	Trial 2		−0.4360	−5.5930	4.3480	–	−1.8772	−6.6110	0.9348	–

A regression-based analysis was carried out with PROCESS macro on SPSS to calculate the mediation effect of systemic inflammation on neurocognitive functions in hepatitis C patients. Helmert coding was used for the grouping of patients with HBV (d1 = −2/3; d2 = 0), HCV (d1 = 1/3; d2 = −-1/2), and treated HCV with SVR (d1 = 1/3; d2 = 1/2). As shown in supplementary Fig. 1, the regression coefficients of viral hepatitis diagnosis on inflammatory markers (ESR or CRP) and the inflammatory markers on neurocognitive functions were calculated and designated as a and b, respectively. Bootstrap confidence intervals for the effects of chronic hepatitis C on the neurocognitive functions through ESR or CRP (ab) based on 5,000 bootstrap samples were calculated to examine whether the effects were significantly different from 0 (both upper and lower limit confidence intervals above or below 0).

*LLCI* lower limit confidence interval, *ULCI* upper limit confidence interval; Sig.: significant; *Statistically significant.

## Discussion

Previous studies rarely investigated the neurocognitive deficit of individuals with chronic HBV or HCV infection in parallel with a detailed neuropsychological test battery covering all the major neurocognitive domains. In the present study, we found neurocognitive deficits in both individuals with chronic HBV and chronic HCV with the comprehensive neuropsychological test battery, and the deficits seem to have different patterns. Patients with chronic HBV were found to have significantly worse performance in verbal comprehension index, information, and matrix reasoning of WAIS-IV than the healthy control group and worse language function assessed with MMSE than the HCV group. These results suggest that chronic HBV causes a neurocognitive deficit in the domains of language function and executive function. Although the negative effect of chronic HBV infection on neurocognitive functions has never been definitely reported, the extrahepatic manifestation of HBV involving the central nervous system has been suggested by previous studies. A retrospective cohort study reported the association between HBV and Parkinson's disease (PD), with a standardized rate ratio of PD following HBV infection found to be 1.76 based on 44 observed compared with 26 expected cases^7^. In one study applying single-cell laser capture methods to assess the full range of microglia-specific expression, HBV was found to be expressed in the microglia. Besides, with immunohistochemical analysis using HBV-core antibody as HBV-positive control, HBV immunoreactivity was found to be increased from amnestic mild cognitive impairment to HBV-positive AD cases as disease pathology increased^8^.

Moreover, in one study investigating the influence of HCV on cognition where HBV was recruited as the control group, the impairment was similar between HBV and HCV in verbal learning/memory when assessed with Rey Auditory Verbal Learning Test^9^. One recent study showed the progressive impairment of dynamic functional network connectivity in patients with HBV-related cirrhosis, although whether the impairment is due to HBV or cirrhosis remains unknown^10^. These previous studies suggest the detrimental impact of HBV on CNS degeneration. The current study demonstrates the effect of chronic HBV infection on neurocognitive functions, with a deficit in language and executive functions.

In addition to the detrimental impact of chronic HBV on language and executive functions, the current study also found that patients with chronic HCV infection showed poorer performance in the tasks associated with executive functions, psychomotor speed, memory, and attention, while visuospatial and language functions remain unaffected. Previous studies had attempted to investigate the impact of HCV on neurocognitive functions, but the findings diverged and varied across different studies. Several studies showed worse performance in tasks associated with attention and executive functions in HCV patients^11,12^, while other studies found the detrimental effect in tasks associated with memory and psychomotor speed^9,13–15^. There were also several studies revealing no definite changes in neurocognitive functions that could be attributable to HCV^16–18^. The conflicting results across the different studies may be due to the difference in the participants' demographic characteristics (such as age and education levels) and the different sensitivity in the tasks used to assess neurocognitive functions.

Moreover, although common comorbidities associated with HCV significantly influence neurocognitive functions, few studies have excluded the patients with depression, cirrhosis, thyroid disorders, cerebrovascular diseases, and end-stage renal diseases in one study to examine the direct impact of HCV on neurocognitive functions^19^. Meanwhile, HCV has also been shown to increase the risk of kidney injury^20^, thyroid disorder^21^, and cerebrovascular events^22^, all of which are associated with neurocognitive impairment. In the current study, to investigate the direct impact of chronic HCV infection on neurocognitive function, we excluded the participants with a history of cerebrovascular disease, brain surgery, thyroid disorder, end-stage renal disease, signs suggestive of decompensated liver cirrhosis, psychiatric disorders, and illicit recreational drug usage. Our results showing the worse performance in tasks associated with executive functions, psychomotor speed, memory, and attention suggest the direct impact of chronic HCV infection on cognition.

We also compared the neurocognitive functions of patients with chronic HCV and those successfully treated and obtained SVR. In the current study, significant improvement in the ALT level, but not the neurocognitive function profiles, was noted, suggesting that the successful elimination of chronic HCV caused improvement in liver function but not neurocognitive functions. However, the results do not necessarily rule out the potentially beneficial effect of successfully eliminating HCV in decreasing the neurocognitive decline rate after the successful treatment. Long-term follow-up is needed to understand the beneficial impact of HCV treatment on neurocognitive function.

Furthermore, the current study found systemic inflammation seems to partially mediate the neurocognitive deficit of individuals with chronic HCV. The significant mediation effect of ESR on the performance in tasks associated with attention suggests that chronic HCV patients' attention deficit is mediated by systemic inflammation. Several mechanisms have been proposed for the neurocognitive deficit associated with HCV. One proposed mechanism suggests that HCV infects the microglia/macrophages in the central nervous system, and the release of proinflammatory cytokines from the infected microglia/macrophages results in neurocognitive deficits^23^. Moreover, as systemic inflammation has been suggested to cause neurocognitive deficits^24–26^, systemic inflammation induced by HCV was also proposed to be involved in the decline of neurocognitive functions. However, no direct evidence has been provided except for the current study^27^. Besides, the mediation effect of ESR on the cognitive domains of attention revealed in the present study may also be in agreement with the pathogenic mechanisms of chronic hepatitis as immune complex diseases^28,29^. Last but not least, HBV- and HCV-infected individuals had been shown to have dysbiosis and change in the composition of intestinal bacteria^30–33^, and the inflammatory responses induced by the translocated microbial products, such as lipopolysaccharide (LPS), were proposed to be one of the pathogenic mechanisms associated with HBV or HCV infection^34^. The association between LPS and the inflammation-mediated attention deficit observed in this study may need further investigation to clarify the liver-gut-brain association.

Meanwhile, CRP was not shown to mediate the neurocognitive dysfunction found in HCV patients in the current study. The dissociation between ESR and CRP in mediating the detrimental effect of HCV suggests the decline in attention associated with HCV is more closely related to the inflammatory conditions that cause elevation of ESR without elevation of CRP. One condition for such dissociation was observed in autoimmune diseases^35^ when interferon-α suppresses the release of CRP. Whether the decline in attention induced by HCV is also associated with pathways related to interferon-α needs further investigation.

Also noteworthy is the difference in the profiles of the neurocognitive function impairment of chronic HBV and chronic HCV patients. While chronic HBV causes deficits in the domains of language and executive functions, chronic HCV was found to cause functional decline in executive functions, psychomotor speed, memory, and attention. The different deficit profiles revealed in the neuropsychological tests may also reflect the potential difference in the mechanisms causing the neurocognitive impairment in chronic HBV or chronic HCV patients. Based on previous studies' results, the deficits found in chronic HBV patients in the language functions in the current study may be associated with cortical dementia syndromes. In contrast, chronic HCV patients' deficits in attention and memory may be more closely related to subcortical dementia syndromes^36^. The results suggest the diverging pathogenic mechanisms of chronic HBV and HCV on neurocognitive functions. In the present study, we focused on the long-term effect of the chronic infections of HBV and HCV on neuropsychological function decline. The neurocognitive function declines found in the chronic HBV and HCV patients and the mediation effect by systemic inflammation shown in the present study suggest a gradual process, in which the waxing and waning disease activity of chronic viral hepatitis causes systemic inflammation that drives the neurodegeneration.

The present study has potential limitations. The significant differences in age and sex ratio between the study groups may cause selection bias. The difference in the education years may interfere with the results of the neurocognitive profile assessment. However, the difference may be inevitable and may reflect the difference in the epidemiological profile of chronic HBV and HCV among Taiwanese, with the prevalence of HBV and HCV in Taiwan being higher in males and females, respectively^37–39^. More importantly, we have used Quade's test to eliminate the effect of the potential confounding factors. The other limitation of the current study is the lack of the laboratory results of ESR and CRP of the healthy control group, which prevents the detailed exploration of the mechanism for the cause of the neurocognitive deficit induced by viral hepatitis, especially HBV. The lack of the healthy control group's laboratory results also prevented the exclusion of patients with end-stage renal diseases and thyroid disorders and may underestimate the detrimental impact of viral hepatitis. However, such underestimation would not cause a significant difference in the main results of the present study.

The other limitation of this study is the lack of laboratory results confirming the absence of human immunodeficiency virus in the participants recruited due to the strict regulations in screening human immunodeficiency virus (HIV) in Taiwan to prevent confidentiality breaches. As the detrimental impact of HIV on neurocognitive functions is significant and well-established^40,41^, and the coinfection of HIV and viral hepatitis is frequent in some areas around the world^42^. However, the transmissions of HBV and HCV in the Taiwanese generation older than 50 years old were mainly due to vertical transmission and iatrogenic factors, respectively^4,5^, while the major risk groups for HIV are those with a history of illegal intravenous drug usage or homosexual practices with men. The prevalence of HIV in the participants recruited in the present study should be low, especially after excluding those with a history of illegal recreational drug usage or same-sex relationship or those with abnormalities suggestive of HIV coinfection during the evaluation by physicians, based on history taking, physical examinations, and laboratory examinations during the clinical follow-up. Therefore, the impact of potential HIV coinfection may be minor, although such a possibility could not be totally excluded in the present study.

Besides, the wide availability of direct-acting antiviral agents against HCV in Taiwan has significantly reduced the number of untreated HCV patients recruited in the present study. Meanwhile, the strict inclusion criteria for participant recruitment in the present study to minimize confounding factors limited the number of participants in the current study and precluded the comparison of the neurocognitive functions across the different disease spectrums. However, the sample size did not cause restriction in the power detecting the neurocognitive deficits and the potential mechanism found in the present study, which focuses on the long-term effect of the chronic infections of HBV and HCV. Future studies with a larger sample size are warranted to validate the detrimental impact of chronic HBV and HCV infection and compare the difference in the neurocognitive deficit conferred across different disease spectrums.

In conclusion, the present study found the detrimental effect of chronic HBV on language and executive functions and showed the negative impact of chronic HCV on executive functions, psychomotor speed, memory, and attention. Also, the neurocognitive function deficit was not reversible after obtaining SVR. Besides, systemic inflammation was found to mediate the deficits in attention in chronic HCV patients. The present study enables a more in-depth understanding of the extrahepatic manifestations of viral hepatitis and also provides evidence for the potential mechanisms of the neurocognitive function deficit induced by viral hepatitis. Lastly, the findings will also raise the awareness of the detrimental impact of viral hepatitis, especially the previously unknown HBV, on neurocognitive functions.

## Methods

### Participants and assessment of clinical presentations

This study cross-sectionally recruited a total of 199 participants, including 39 healthy volunteers, 72 patients diagnosed with HBV, and 88 patients diagnosed with HCV between the 21^st^ of September 2017 to the 24th of March 2020. Out of the 88 HCV patients, 65 have obtained SVR for more than six months before recruitment in the study. Among the 23 participants without SVR, 22 participants were treatment naïve except for one participant who had received peginterferon and ribavirin 17 years ago. Among the 65 participants obtaining SVR, 35 obtained SVR with peginterferon and ribavirin, and 30 obtained SVR after direct-acting antiviral agents. The decision to initiate antiviral therapy for HBV was primarily based on the presence or absence of cirrhosis, the ALT level, and the HBV DNA level following the EASL 2017 guidelines for HBV infection^43^. All participants were between 40 and 80 years old with an education level equal to or more than six years to fully understand the content of the neuropsychological assessment. We excluded patients with a history of cerebrovascular disease, brain surgery, thyroid disorder, malignancy, alcohol abuse, end-stage renal disease, signs suggestive of decompensated cirrhosis (including ascites, jaundice, esophageal variceal bleeding, or hepatic encephalopathy), psychiatric disorders, and current pregnancy or breastfeeding. We also excluded patients with signs suggestive of organic brain lesions after a neurological examination by a board-certified neurologist, including 1 individual with HBV, 2 individuals with untreated HCV, and 6 individuals with treated HCV before the recruitment. Participants with HIV or a history of illicit recreational drug usage and tattooing were excluded to minimize the possibility of recruiting patients with HIV in the study. Meanwhile, individuals in same-sex relationships were also not recruited. Participants with end-stage renal disease were excluded based on the estimated glomerular filtration rate < 30 mL/min/1.73m^2^ calculated with CKD-EPI Creatinine Equation. The exclusion of participants with decompensated cirrhosis was based on history taking and clinical assessment by the hepatologists referring the patients.

The participants' written informed consent was obtained before enrollment, following the ethical standards outlined in the 1975 Declaration of Helsinki. All study procedures were approved by the ethical research committee of Kaohsiung Medical University Hospital (KMUHIRB-G(I)-20170022), and National Cheng Kung University Hospital (-/B-ER-104-082), and all methods were performed following the approved guidelines.

### Neurological examinations

All participants in the disease groups were assessed with a neurological examination. The neurological examination performed by a board-certified neurologist is composed of the examinations of cranial nerves, motor functions, sensory functions, coordination, and reflexes. Participants with signs suggestive of organic brain lesions were not recruited for further evaluation to exclude the presence of stroke^44,45^ or intracranial lesions^46^ that are associated with viral hepatitis. Participants with signs suggestive of peripheral nervous system disorders were not excluded as the degeneration of the peripheral nervous system and the neurocognition may share common pathogenic mechanisms^47^.

### Neuropsychological assessment

All study participants were assessed with a comprehensive neuropsychological battery composed of tests evaluating all the major neurocognitive domains, including memory, psychomotor speed, attention, visuospatial, executive function, and language function, similar to our previous investigation on the cognitive profiles of patients with PD^48^. The disease state of chronic viral hepatitis and the state of sustained virologic response had been confirmed with laboratory tests before the evaluation of neuropsychological assessment. Logical Memory-I&II and Visual Reproduction-I&II in the WMS-III were used to assess memory^49^. Trial 1 of CTT^50^, Digit Symbol Substitution, and Symbol Search were used for psychomotor speed evaluation. Stroop Color and Word Test^51^, Digit Span of WAIS-IV^52^, PASAT^53^, and serial seven of MMSE^54^ were used to evaluate attention. Block Design of WAIS-IV was used for visuospatial function evaluation. Similarities, Matrix Reasoning, Information, Letter-Number Sequencing of WAIS-IV, Semantic Association of category verbal fluency^55^, trial 2 of CTT, and M-WCST^56^ were used to assess executive function. Naming, repetition, verbal comprehension, and writing of MMSE were used for language function evaluation. The raw scores of the neuropsychological tests were used for the analysis. The neuropsychological tests used for the assessment of the major neurocognitive domains are summarized in Table 1.

### Laboratory evaluation

The diagnosis of HBV was based on the serological test positive for HBsAg, and the diagnosis of HCV was based on the serological test positive for the anti-HCV antibody. The presence of the HCV virus in the HCV patients was confirmed with polymerase chain reactions. The laboratory tests used to diagnose viral hepatitis were collected between the 11th of April 2001 and the 8^th^ of May 2019. The creatinine levels of all the participants in the study group of HBV, HCV, or SVR were also examined to rule out the possibility of neurocognitive impairment attributable to end-stage renal disease. Levels of the ALT of chronic hepatitis patients were examined to evaluate their liver function. In addition, aspartate transaminase (AST) and platelet count of chronic hepatitis patients were examined to obtain the FIB-4 index, as an indicator of cirrhosis. Levels of ESR and CRP were also examined among the participants with HBV, HCV, or SVR to evaluate systemic inflammation status. The laboratory examinations for ALT, creatinine, ESR, and CRP were collected within the one-month period from the date of neuropsychological tests. The date of the sample collection for the laboratory results used to calculate FIB-4, including AST, ALT, and platelet count, was within the six-month period from the date of neuropsychological tests. There were two participants in the SVR group whose ESR and CRP were not examined.

### Statistical analysis

Similar to our previous publication^57^, proportions were calculated for qualitative variables, and means and standard deviations were calculated for quantitative variables. We used the Kolmogorov–Smirnov test for the normality test. Afterward, we examined quantitative variables with the Kruskal–Wallis test and qualitative variables with a chi-square test. We controlled the impact of confounding variables (age, sex, and education years) with Quade's test^58^. Statistical significance was predetermined with an alpha level less than 0.05. The statistical analysis was performed by a commercially available software program (IBM Corp. Released 2012. IBM SPSS Statistics for Windows, Version 21.0. Armonk, NY: IBM Corp.). A regression-based analysis was carried out with PROCESS macro on SPSS^59^ to calculate the mediation effect of systemic inflammation on neurocognitive functions in hepatitis C patients. Helmert coding was used for the grouping of patients with HBV (d1 = −2/3; d2 = 0), HCV (d1 = 1/3; d2 = −1/2), and treated HCV with SVR (d1 = 1/3; d2 = 1/2). Bias-corrected bootstrap confidence intervals for the effect based on 5000 bootstrap samples were calculated and used to determine whether ESR or CRP mediated the neurocognitive function changes in hepatitis C patients. All the associated data not provided within the paper are available on request from Rwei-Ling Yu.

## Supplementary Information

Supplementary Information.
