# Supplementary material for: Different profiles of neurocognitive impairment in patients with hepatitis B and C virus infections
Source: Sci Rep. 2022 Jun 23;12:10625. doi: 10.1038/s41598-022-14736-3 (PMC9226189; doi:10.1038/s41598-022-14736-3)
Supplement: Supplementary file 1 — Supplementary Information. [file 41598_2022_14736_MOESM1_ESM.pdf]

Supplementary Information

Different Profiles of Neurocognitive Impairment Caused by Hepatitis B and C Virus Infections

Chun-Hsiang Tan [1,2], Meng-Chia Chang [3], Wei-Fang Tsai [4], Wan-Long Chuang [5], Jee-Fu Huang [5], Zu-Yau Lin [5], Chia-Yen Dai [5], Ming-Lun Yeh [5], Chi-Ting Li [6], Rwei-Ling Yu [3]*

[1] Department of Neurology, Kaohsiung Medical University Hospital, Kaohsiung Medical University, Kaohsiung, Taiwan

[2] Graduate Institute of Clinical Medicine, College of Medicine, Kaohsiung Medical University, Kaohsiung, Taiwan

[3] Institute of Behavioral Medicine, College of Medicine, National Cheng Kung University, Tainan, Taiwan

[4] M.Sc. Program in Tropical Medicine, College of Medicine, Kaohsiung Medical University, Kaohsiung, Taiwan

[5] Hepatobiliary Division, Department of Internal Medicine, Kaohsiung Medical University Hospital, Kaohsiung Medical University, Kaohsiung, Taiwan

[6] Department of Psychology, Kaohsiung Medical University, Kaohsiung, Taiwan

Supplementary table 1. Comparison of ESR and CRP between the study groups with age and sex corrected.

	HBV-HCV		HBV-SVR		HCV-SVR	
	Quade's test					
	F	P	F	P	F	P
ESR	3.483	0.065	0.822	0.366	0.927	0.338
CRP	0.227	0.635	0.815	0.369	0.994	0.322

Abbreviation used in this table: CRP: C reactive protein; ESR: erythrocyte sedimentation rate; HBV: hepatitis B virus; HCV: hepatitis C virus; SVR: sustained virologic response

Supplementary Table 2. Post-hoc comparison of neurocognitive functions between the study groups with Dunn's test.

			Control-HBV	Control-HCV	Control-SVR	HBV-HCV	HBV-SVR	HCV-SVR
			Post-hoc with Dunn's test					
			P	P	P	P	P	P
**Mini Mental Status Examination**	Serial 7		-	-	-	-	-	-
	Language		-	-	-	-	-	-
**Wechsler Memory Scale-III**	Logical Memory	Immediate recall	1.000	1.000	0.296	0.559	0.049*	1.000
		Delayed recall	1.000	1.000	0.205	0.874	0.010*	1.000
	Visual Reproduction	Immediate recall	1.000	1.000	0.018*	1.000	0.031*	1.000
		Delayed recall	1.000	0.350	0.832	0.131	0.240	1.000
**Wechsler Adult Intelligence Scale-IV**	Full Scale Intelligence Quotient		1.000	0.020*	0.026*	0.080	0.125	1.000
	Verbal Comprehension Index		0.782	0.003*	0.001*	0.061	0.044	1.000
	Perceptual Reasoning Index		1.000	0.009*	0.023*	0.060	0.205	1.000
	Working Memory Index		-	-	-	-	-	-
	Processing Speed Index		-	-	-	-	-	-
	Similarities		1.000	0.004*	0.008*	0.005*	0.009*	1.000
	Information		1.000	0.046*	0.036*	0.080	0.055	1.000
	Block Design with No Time Bonus		1.000	0.066	0.506	0.014*	0.098	1.000
	Matrix Reasoning		1.000	0.153	0.107	0.631	0.614	1.000
	Letter-Number Sequencing		-	-	-	-	-	-
	Digit Span		1.000	0.565	0.969	0.030*	0.015*	1.000
	Longest Digit Span Forward		0.198	0.612	1.000	0.002*	0.012*	1.000
	Longest Digit Span Backward		-	-	-	-	-	-
	Digit Symbol Substitution		0.396	1.000	1.000	0.269	0.040*	1.000
	Symbol Search		0.185	1.000	1.000	0.051	0.362	1.000
**Paced Auditory Serial Addition Test**			0.898	0.916	1.000	0.036*	0.206	1.000
**Modified Wisconsin Card Sorting Test**	Number of Categories Completed		-	-	-	-	-	-
	Number of Perseverative Errors		-	-	-	-	-	-
**Stroop Color and Word Test**	Interference Score		-	-	-	-	-	-
**Semantic Association of Category Verbal Fluency**	Total		0.032*	1.000	0.827	0.340	0.855	1.000
**Color Trails Test**	Trial 1		-	-	-	-	-	-
	Trial 2		0.085	1.000	1.000	0.121	0.086	1.000

Abbreviation used in this table: HBV: hepatitis B virus; HCV: hepatitis C virus; SVR: sustained virologic response

Supplementary table 3. Mediation analysis of neurocognitive functions between the study groups by CRP

			Mediation Analysis							
			No Covariates				Adjusted for age, sex, education and metabolic syndrome			
			effect	LLCI	ULCI	Sig.	effect	LLCI	ULCI	Sig.
**Mini Mental Status Examination**	Serial 7		-0.0513	-0.2333	0.0291	-	-0.0480	-0.2087	0.0236	-
	Language		0.0043	-0.0137	0.0222	-	0.0034	-0.0129	0.0231	-
**Wechsler Memory Scale-III**	Logical Memory	Immediate recall	-0.3552	-1.4521	0.2898	-	-0.3661	-1.3937	0.2191	-
		Delayed recall	-0.1668	-0.6661	0.2297	-	-0.1801	-0.6912	0.1813	-
	Visual Reproduction	Immediate recall	0.3620	-0.5298	1.1451	-	0.2942	-0.3649	1.0104	-
		Delayed recall	0.3314	-1.0481	1.1883	-	0.2596	-0.8547	1.1321	-
**Wechsler Adult Intelligence Scale-IV**	Full Scale Intelligence Quotient		-0.3234	-1.8758	0.3532	-	-0.2967	-1.1975	0.2983	-
	Verbal Comprehension Index		-0.3228	-2.1025	0.2988	-	-0.2829	-1.1273	0.1722	-
	Perceptual Reasoning Index		-0.1569	-1.0010	0.2653	-	-0.1152	-0.5418	0.3758	-
	Working Memory Index		-0.4035	-1.9778	0.2154	-	-0.3849	-1.5387	0.2677	-
	Processing Speed Index		-0.3058	-1.9144	0.5488	-	-0.2920	-1.3814	0.4794	-
	Similarities		-0.1527	-0.7633	0.0905	-	-0.1493	-0.5393	0.0731	-
	Information		-0.0468	-0.5489	0.1373	-	-0.0457	-0.3024	0.0895	-
	Block Design with No Time Bonus		-0.1549	-0.4807	0.1758	-	-0.1356	-0.3602	0.1440	-
	Matrix Reasoning		0.0994	-0.1324	0.3905	-	0.0808	-0.0430	0.3256	-
	Letter-Number Sequencing		-0.1211	-0.6497	0.1774	-	-0.1256	-0.6026	0.1676	-
	Digit Span		-0.0520	-0.3339	0.0661	-	-0.0672	-0.2900	0.0593	-
	Longest Digit Span Forward		-0.0148	-0.0829	0.0141	-	-0.0150	-0.0672	0.0166	-
	Longest Digit Span Backward		-0.0254	-0.1229	0.0212	-	-0.0253	-0.1058	0.0208	-
	Digit Symbol Substitution		0.2969	-0.8291	2.3529	-	0.1936	-0.8382	1.6877	-
	Symbol Search		-0.0990	-0.5823	0.2787	-	-0.1274	-0.6220	0.2271	-
**Paced Auditory Serial Addition Test**			-0.2809	-1.4050	0.9644	-	-0.2880	-1.3436	0.9932	-
**Modified Wisconsin Card Sorting Test**	Number of Categories Completed		-0.0031	-0.0498	0.1423	-	-0.0080	-0.0634	0.1073	-
	Number of Perseverative Errors		-0.0115	-0.2623	0.1138	-	0.0000	-0.2451	0.1368	-
**Stroop Color and Word Test**	Interference Score		-0.1632	-0.9472	0.2290	-	-0.1589	-0.8098	0.1939	-
**Semantic Association of Category Verbal Fluency**	Total		-0.2295	-0.7236	0.2340	-	-0.2374	-0.8269	0.1172	-
**Color Trails Test**	Trial 1		-0.0527	-1.3242	0.3318	-	-0.0009	-1.0670	0.4468	-
	Trial 2		0.0396	-1.9136	0.9081	-	0.1022	-1.6539	0.9808	-

A regression-based analysis was carried out with PROCESS macro on SPSS to calculate the mediation effect of systemic inflammation on neurocognitive functions induced by hepatitis C. Helmert coding was used for the grouping of patients with HBV (d1=-2/3; d2=0), HCV (d1=1/3; d2=-1/2), and treated HCV with SVR (d1=1/3; d2=1/2). As shown in

supplementary figure 1, the regression coefficients of viral hepatitis diagnosis on inflammatory markers (CRP) and the inflammatory markers on neurocognitive functions were calculated and designated as a and b, respectively. Bootstrap confidence intervals for the effects of chronic hepatitis C on the neurocognitive functions through CRP (ab) based on 5,000 bootstrap samples were calculated to examine whether the effects were significantly different from 0 (both upper and lower limit confidence intervals above or below 0).

Abbreviation used in this table: LLCI: lower limit confidence interval; ULCI: upper limit confidence interval; Sig.: significant.

Supplementary figure 1

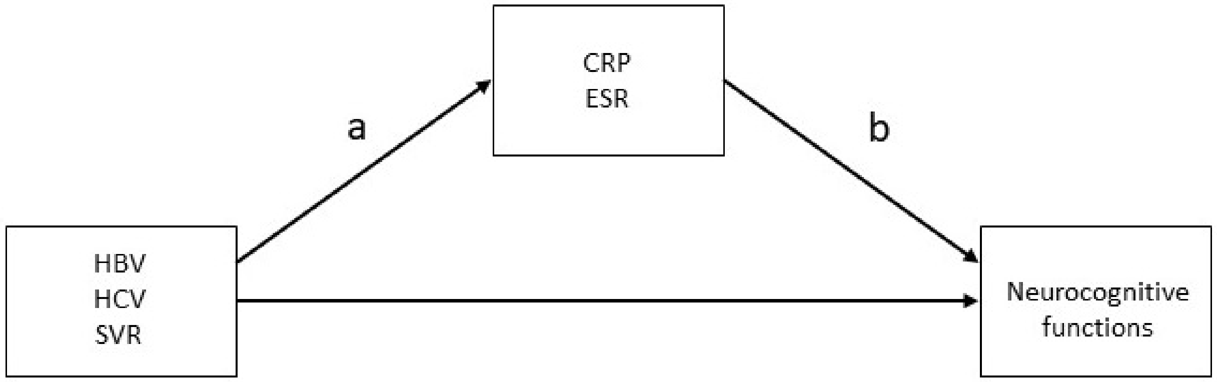

Supplementary figure 1. A statistical diagram of the simple mediation model illustrating the calculation of the mediation effect of systemic inflammation on neurocognitive functions induced by hepatitis C.
